# Hospital and Institutional News

**Published:** 1914-01-10

**Authors:** 


### HOSPITAL AND INSTITUTIONAL NEWS.

### THE NEW YEAR HONOURS.

THERE are twelve names of interest to the institutional and medical world which are included in the list of New Year honours. In the first place, to take them in the order of their distinctions, Sir Christopher Nixon, Bart., Professor of Medicine at the Catholic University of Ireland, and former P.R.C.P. of Ireland, has been made a member of the Privy Council in Ireland. Sir Christopher is senior physician to the Mater Misericordiæ Hospital. There is only one medical baronet, namely, Mr. Thomas Joseph Stafford, F.R.C.S., Medical Commissioner of the Local Government Board. There are no medical knights, but the institutional world is honoured in the knighthood which has been conferred upon Mr. W. James Thomas, the munificent supporter of the Cardiff Medical School and King Edward VII.'s Hospital, Cardiff, to whose benefactions we have made frequent allusions in these columns, and in that conferred on Mr. R. B. D. Acland, former chairman of the Hospital Saturday Fund, who has done so much for hospital work in London. In the military division of the Order of the Bath a C.B. has been conferred upon Surgeon-General Harold George Hathaway, Deputy Director of Medical Services in India, and in the Colonial Office List the honour of Knight Bachelor has been bestowed on Mr. Harry Brookes Allen, M.D., who is Professor of Pathology and Dean of the Faculty of Medicine at the University of Melbourne. Dr. Allen has contributed several important reports to the Victorian Government on hospital construction, and also on tuberculin. Before glancing at the Indian List two inevitable distinctions must be mentioned, namely, the K.C.V.O. which is conferred upon Sir Rickman Godlee, President of the Royal College of Surgeons, and Sir William Collins, one of the few medical men who has consistently taken an active part in public and Parliamentary life, by his legislative efforts in the House of Commons and on the London County Council. Lastly, the C.I.E. is bestowed on Major James Currie Robertson, M.B., Sanitary Commissioner with the Government of India, while knighthoods on the Indian List are given to Dr. Temulji Bhikaji Nariman, a medical practitioner of Bombay, and to Surgeon-General Arthur Thomas Sloggett, C.B., Director of Medical Services in India.

### A HOSPITAL KNIGHT.

THE honour of knighthood conferred by the King upon Mr. William James Thomas, of Ynyshir, as we note above, has given great satisfaction to all classes of people in South Wales, and especially to those associated with its hospitals. His benefactions to the King Edward VII.'s Hospital, Cardiff, have been princely, and the "W. James Thomas" Ward in that institution is proudly regarded in Cardiff as one of the most beautiful and best-equipped in Europe. The realisation of the Cardiff Medical School will owe much to his generosity, as he has already pledged himself to an expenditure approaching £30,000 for the erection and equipment of a complete Physiological Block. Recently, the Royal Gwent Hospital at Newport has received from Sir William Thomas very substantial donations. We have the best authority for stating that his little acts of kindness often exact from him a greater personal care than his great gifts, though these frequently run into four figures—and occasionally into five. King Edward VII.'s Hospital, Cardiff, has amongst its pleasantest experiences and recollections Sir William Thomas's personal care and thought for the patients with whom he has been brought into contact, either in the coalfield or at the hospital. Amongst the numerous congratulatory messages sent to him none will be more highly valued than the telegram sent, on their own initiative, by the patients of the "W. James Thomas" Ward.

### HONOUR FOR MEDICAL MISSIONS.

THE Kaisar-i-Hind Medal for Public Service in India has been awarded to Dr. William Stokes, M.B., chief of the Basel German Medical Mission, Madras, to Dr. Marcus Bradford Carleton, M.D., who is in charge of the Leper Asylum at Subathu, Punjab, and to the Rev. Paul Wagner, superintendent of the Leper Asylum, Purulia, Bihar and Orissa, and to the Rev. James Shepherd, M.D., D.D., of the Medical Mission of the United Free Church of Scotland, Udaipur, Rajputana. Anything which emphasises the supreme importance of medicine and medical training in the mission field is bound to be welcome, and the above honours are the most interesting feature of the Indian and Colonial Lists.

### MEDICAL WITNESSES IN CORONERS' COURTS.

TEN days ago the senior surgeon at Blackburn Infirmary, Dr. Core, was severely reprimanded by the Coroner, Mr. D. N. Haslewood, for being late at an inquest held at the institution. Dr. Core said that he came as soon as he heard that he was wanted. The Coroner retorted that it was not the first time they had had to wait for medical witnesses, and threatened to report the matter. Thereupon a solicitor, Mr. Backhouse, followed the lead given to him, and turned on Dr. Core with the remark, "Our time is much too valuable to be kept waiting here." To this Dr. Core quietly replied that he had been assisting at an operation. He might truly have said also that so long as the law refuses to regard the time of medical witnesses attached to institutions as worthy of a fee for attendance at Coroners' Courts, so long will they be tempted to regard Coroners' and solicitors' time as equally unworthy of consideration. We do not believe that Dr. Core, as a matter of fact, felt, much less yielded to, this temptation, but the Coroner and the solicitor were only adding insult to injury by adopting the abominably rude attitude mentioned above.

### NEW MENTAL MEDICAL OFFICERS' ASSOCIATION.

We are officially informed that the new Association of Assistant Medical Officers of Mental Hospitals in England and Wales has elected the following officers: Dr. E. L. Dunn, B.A., M.B., B.Ch.Dublin, the County Mental Hospital, Wallingford, Berkshire, has been appointed chairman; Dr. W. Maule Smith, M.D., of Barnsley Hall Mental Hospital, Bromsgrove, Worcestershire (2 Victoria Road, Bromsgrove), has been appointed honorary treasurer; and Mr. F. J. Stuart, M.R.C.S., L.R.C.P., of Berry Wood, Northampton, has been appointed honorary secretary.

### LOCK HOSPITALS LEFT IN THE LURCH.

Philanthropists in search of something to endow as urgent and more neglected than cancer, consumption, or tropical research may well turn their attention to the plea of the London Lock Hospital for funds to extend the salvarsan treatment already given within the limits of financial possibility to patients of that institution. Mr. J. F. W. Deacon, the treasurer of what he proudly describes as "the only hospital in London" for the sole treatment of venereal diseases, points out that £10,000 is required if the extended treatment contemplated by the board is to be carried out. In spite of the fact that the male Lock Hospital has been in process of rebuilding for the past two years, 900 salvarsan injections have been given, and now that the building is complete, he adds that they are being administered at the rate of from forty to fifty a week. At the Female Lock Hospital 423 injections were given last year. The special departments for treating the disease at the general hospitals cannot be adequately organised on the lines laid down by Ehrlich in a moment, and the stream of charity, which recent events have markedly shown to be flowing in full vigour towards the voluntary hospitals, might well be diverted towards the endowment of the newer treatments of syphilis. Unfortunately, it is much easier to throw the forces of philanthropy into a new channel than to recall them when they have gone far enough; but surely some man of wealth must have intelligence enough to realise that other lines of research and treatment besides the three favourites mentioned above demand his attention and support. The fact of the appointment of a Royal Commission should be enough to prove that here is a need newly given public consideration which deserves, perhaps better than any, the service of wealth and sagacity.

### NIGHT CLINICS AND THE LONDON LOCK HOSPITAL.

In some interesting evidence before the Royal Commission on Venereal Diseases Mr. J. Ernest Lane, who is a member of the Commission, and senior surgeon of the London Lock Hospital, said that for efficient treatment night clinics were essential, but that he thought notification would deter patients from all but quack treatments, which it might encourage. He added the appalling statement, however little surprising it is to those who are acquainted with the facts, that 13 per cent. of the patients at the London Lock Hospital were married women, and all the cases in the children's wards were examples of innocent syphilis. Such facts should be enough finally to dispose of the monstrous argument that the disease punishes only those people who are guilty, and this, indeed, is shown once again to be nothing but an excuse for shutting our eyes to the facts. As to notification, we have previously shown that any system of notification to succeed must be an impersonal one, and there seems no reason why an impersonal system could not be devised in this country. It is noteworthy that the arts of literature and the drama have been the pioneers in breaking down the taboo that has hedged this subject, and this fact illustrates the argument of artists in all departments of letters that censorship is against the public good, since public evils must be exposed publicly if the exposure and the knowledge bred by its means are to be exposures in fact as well as in name. In truth, if the Royal Commission is not to be our characteristic way of shelving questions which there is sufficient public opinion to force into the field of public life, the arts must continue to support the movement for public action which the institutional world may gladly acknowledge they have done so much to bring into being.

### POST-GRADUATION LECTURES ON CHILDREN'S DISEASES.

A further course of Post-Graduation Lectures is being given at the Queen's Hospital for Children, Hackney Road. The lectures, which will be delivered at 4 P.M. in the ward-room during January, February, and March, will be free to all medical practitioners and students of medicine, and deal with such varied subjects as spinal caries, orthopædic cases, anæmia in childhood, epilepsy, illustrated by cases. Dr. E. Bellingham Smith, honorary secretary of the medical committee, will be pleased to supply all further particulars.

### AMBULANCE HOSPITALS IN SOUTH WALES.

The Cardiff newspapers have again begun to discuss the need for hospital accommodation at the pit-heads of the collieries, and instance the stations that the St. John Ambulance Association have erected here and there as examples of the practicability of the idea. The Great Western Colliery, Pontypridd, and some dozen other pits similarly furnished are mentioned, together with the Central Store of first-aid equipment at the headquarters of the Welsh District Prudential Buildings, Cardiff. It is also stated that the workmen at the Plymouth and Cyfarthfa Collieries have agreed to contribute 6d. per man per annum, and 3d. per boy per annum, to the expenses of the St. John Ambulance Brigade in that district, over which a general committee will preside to apportion the money and to represent the various divisions. Moreover, the latest benefaction of Sir W. J. Thomas, whose knighthood we refer to in another note, appears to be a conditional gift of £1,000 for a new ambulance, provided £2,000 is forthcoming to build a central ambulance stores in Cardiff, from which an ambulance and all equipment may be instantly despatched to any part of the coal-field. Admirable as this movement is, there is an alternative to an ambulance station—namely, the plan for emergency hospitals, which was described and illustrated with plans in The Hospital of June 22, 1912 (page 305). The hospital ward there described was estimated to cost less than £200, and the number of deaths would be considerably lessened were this means of combating shock made compulsory at every pit-head and works establishment. Ambulance stations are not enough. Emergency wards are the real need, and, as will be seen, not an expensive one.

### PANEL PRACTICE AND THE POOR-LAW INFIRMARIES.

Serious complaints have been made by the Southwark Board of Guardians that people in the poorer districts have been unable to obtain the services of a doctor in cases of serious illness, although they were willing to pay a small fee. Before the Act came into operation the poor had too many doctors vying with each other to attend them professionally at very low fees. Since the Act the medical man who formerly catered for their needs has sufficient patients on his panel to ensure him a comfortable income. In one case which came under the notice of the Southwark Guardians an aged woman lay insensible in her home for over eight hours, although her son, a respectable working man, had asked four doctors to attend her. Two had promised to do so, but when appealed to a second time excused themselves with the remark that they were too busy attending to their panel patients. The son was reluctantly compelled to call in the parish doctor. Unfortunately, a large number of these cases have occurred, with the result that Poor-Law infirmaries of London have to accommodate a number of patients, who, under the old conditions, would have been attended at home by a private medical man.

### A PANEL DOCTOR'S CIRCULAR.

The Middlesex Local Medical Committee having complained that a panel doctor had issued a certain circular, it was decided at a meeting of the Middlesex Insurance Committee on Monday that, unless he apologised and consented not to issue the circular or any similar to it, the Insurance Commissioners should be informed that his continuance on the panel was prejudicial to the medical service. The circular in question, which was reported by the Medical Service Sub-Committee, read: "National Health Insurance Act, 1911.—All persons having medical tickets should sign them with their full names and addresses, and lose no time in having them signed and legalised. If this is not done, they are unable to draw their sick pay and obtain other advantages. Cards may be legalised at 51 The Avenue, Kew Gardens (opposite the church), after which the Insurance Committee will be notified, and the necessary entries made in their books to ensure the proper benefits being received by the insured member." The Sub-Committee held the circular objectionable as obscuring the right of insured persons to free choice of panel doctors. They recommended his removal from the panel, because he had justified its issue. It would be interesting to know on what grounds the panel doctor justified this remarkable document, and what were the motives which led him to be responsible for drafting it.

### HONOUR FOR A HOSPITAL FOUNDER.

The Oxford Eye Hospital at a special meeting of the general committee has decided, after a lapse of twenty-seven years, to honour in practical form the name of its founder, Dr. Robert Walter Doyne. The specific question submitted to the meeting was to consider how best to connect permanently the name of Robert Walter Doyne with that of the institution, and Mr. Menteith Ogilvie proposed that in all future annual reports, leaflets, and letter headings the words "Founded by Robert Walter Doyne in 1886" be placed immediately below the name of the hospital. This proposal was seconded by Mr. H. M. Dowson, and supported by Sir Frederick Bradshaw, the distinguished honorary secretary, and unanimously carried. No one can possibly quarrel with it, but experienced administrators will feel that such a good opportunity of improving the occasion by adding to the hospital's usefulness should not have been wasted. If there was sufficient feeling to support a project for honouring the name of the founder, there should have been sufficient enterprise to supply some want of funds, equipment, and accommodation. Or does the Oxford Eye Hospital need nothing at the present time?

### ROUTINE AND RESEARCH AT GLASGOW.

The annual report on the work of the clinical laboratory attached to the Western Infirmary, Glasgow, bears witness to the large amount of original research which is carried out there, in addition to the routine of examinations and reports on materials collected in the infirmary by those in medical charge of the patients. To deal first with the latter, it is of interest to note that the Wassermann reaction is that which is most often performed of all the bacteriological clinical tests used in diagnosis. It was carried out 356 times, whereas only 160 throat swabs (for diphtheria) were submitted, 34 samples of blood for Widal's reaction (typhoid fever), and 175 sputa (for tubercle bacilli principally). Besides these, pus was bacteriologically examined 245 times, often for the preparation of vaccines; and urine on 204 occasions. There were, of course, many other investigations of the like nature; but the examples given show the important part which this laboratory plays in the working of the Western Infirmary. Teaching also flourishes, for post-graduate as well as student courses are conducted. Research is even more assiduously practised: the list of papers by the nine research workers in the laboratory, published during last year, is sufficient evidence of this. Such summaries as the Glasgow Western Infirmary sends out might usefully be issued by other hospitals, for subscribers would then realise the return that is got for the money sunk in laboratories and their upkeep. We shall look forward with interest to future developments along this line from the Western Infirmary.

### DEATH OF THE INVENTOR OF "REST CURE."

DR. WEIR MITCHELL, physician, novelist, and poet, has died at Philadelphia at the age of eighty-four. Known to fame in the three capacities here mentioned, he received his medical education at Jefferson Medical College, Philadelphia, and during the American Civil War superintended Turner's Lane Hospital, which was established for the treatment of nervous shock after the reception of wounds. The war and his experience of the sufferings endured by his patients proved the turning point of his life by directing his attention to diseases of the nervous system, on which in 1882 he published a since famous book, in which he collected evidence to show that hysteria was a disease, and not a form of malingering, and for it he prescribed the now classic "rest-cure" treatment. He wrote also works on toxicology, the effect of such drugs as chloral and opium, an eminently successful novel, published in 1897, entitled "Hugh Wynne, Free Quaker," describing the life and manners of his birthplace at the revolutionary period, and various volumes of poems, including "Francis Drake," a poetic drama, "The Masque," which was staged by the late Wilson Barrett, and numerous other volumes of a medical or purely literary kind. In this dual capacity he followed his father, Dr. John Kearsley Mitchell, who was also a medical man and an author. He was formerly President of the College of Physicians, Philadelphia, and his most permanent achievement was probably the investigation of nervous diseases, which led to his "rest-cure" treatment, which, however, is in danger of doing him less honour than his work deserves from the unscientific exploitation accorded to it from hotels and other commercial undertakings, who naturally like to cloak the cost of laziness with pretended medical sanction.

### INCREASE OF LEPROSY IN FRANCE.

OWING to the increase of leprosy in Paris and in France generally, the Ministry of the Interior has asked Dr. Marchoux, according to the *Times* correspondent, to report on the best preventive methods. There are said to be now 300 lepers in Paris, or six times as many as were known a few years ago, and the disease is not at present notifiable. Of the sewer rats of Paris 5 per cent. are reported as leprous. Dr. Marchoux, writes the same authority, sees no great reason why there should not be another epidemic of mediæval magnitude. Parliament, we learn, will be invited to enforce the notification of the disease and the isolation of lepers, for which possibly special lazar houses may be required. The institutional treatment here proposed will be in the hands of a medical committee authorised to enforce removal from private houses not effectively isolating patients. We hope that Dr. Marchoux's views will prove alarmist merely, but it would be no tribute to science to have to admit that epidemics alone are sufficient to secure adequate investigation and research. It is not wholly thanks to preventive methods scientifically promulgated that leprosy has largely lost its terrors for Europeans.

### WASTE PRODUCTS AS A SOURCE OF INCOME.

THE modern factory system has made its commercial success on the principle of eliminating waste products by finding side uses for substances that are ejected as waste from the main work of the factory. The recognition that there is really no such thing as a waste product—manure being the basis of agriculture, for instance—has now spread so that we are always being invited to save our "cigarette pictures" or soap-wrappers or waste paper, and occasionally enterprising newspapers offer to collect the contents of our dustbins and to hand over the proceeds of their sale to this or that charity. A curious instance of this is reported from Belgium, where, it seems, fifteen years ago the idea began of collecting odd pieces of tinfoil and lead, and selling them for the benefit of hospitals. It is now stated that the society which originated the scheme is now the mainstay of four hospitals for children, whose upkeep, however, is also secured through the ordinary channels of subscriptions. The plan has also been adopted by the Ancient Order of Druids in England, whose Secretary, Mr. L. Du Four, adopted it in 1912, and has sent an aggregate of some £100 to the Middlesex Hospital toward the endowment of a bed. Incredible though it sounds, by the efforts of the society it appears that over 8,500 lb. of tinfoil have been collected and disposed of through the efforts of the society's collectors. In view of the countless odds and ends that must be wasted by us all in this fashion, it seems a pity that the matter is not taken up everywhere by the local authorities, so that the dustman's visit might be welcomed from the knowledge that the rubbish which he removes is going in relief of rates after being sorted and valued at the local refuse store! Till that day comes, rubbish is certainly the legitimate heritage of charity.

### A QUERY ON CHRISTMAS REJOICINGS.

THE Secretary of a large hospital in London writes: "This question of the Christmas entertainment to the staff is a difficult one to tackle. In some hospitals there is no attempt to brighten with reasonable festivity the period of Christmastide, while in others it runs riot. Most hospitals would be glad of a hospital authority here—a guiding hand to some uniformity—a lead as to what is the right thing to do. There is a change for the better during the last decade or two, though it costs more. At one time the idea of a Christmas gathering consisted of a general supper at which the medical and nursing staff, the porters and the maids, all sat at table together—even governors and committeemen used to attend these feasts—aye! and sing, too. All this is only a memory now. Then came the concert and theatricals, with refreshments afterwards, at which everybody was present. Then the much-debated nurses' dance—surely no matter ever gave rise to so much difficulty, so much heartburning. We don't dance now in hospitals; we are above it; a higher tone pervades our revels. The medical officers dine together, and the nursing staff have their own chaste gathering within the sacred precincts of the home, while the ward maids and servants are patronised and given the good things of the season. But where is the line to be drawn? Is the hospital justified in providing champagne and truffle to the housemen and nurses and supplying hospital port to the servants and porters? How does this strike the public? We want a lead or a pronouncement somewhere."

### THE STAFF LADDER AT SWANSEA HOSPITAL.

At a meeting of the board of management of Swansea Hospital last week the house committee and the medical staff recommended that Dr. Clarke Begg be senior assistant physician, Dr. Trevor Evans second assistant surgeon, and Dr. Urban Marks second assistant physician, and by twenty-three votes to eighteen these proposals were carried, the minority being in favour of advertising the vacancies. Encouraged no doubt by the narrow majority against him, Mr. Tuckfield gave notice of a motion to rescind the present custom of allowing members of the medical staff automatically to ascend the ladder, and in future that the vacancies should be advertised. Let him be warned, however, that such advertisements should be properly drafted, since we have often had to call attention in these columns to the practice of certain hospitals, which, while advertising the higher posts, carefully expunge from their advertisements all particulars concerning them, with the aim and result of "warning" readers that the advertisement is only a cloak for a previously determined appointment. Such a step at Swansea would be actually retrograde.

### SALARIES AND THE SATURDAY FUND.

At the quarterly meeting of the Board of Delegates of the Hospital Saturday Fund Mr. Harry Gower presiding, the Secretary, Mr. A. W. Davis, stated that the sum received at the head office to date had amounted to £21,950. There had been fewer claims on the Fund for sanatorium benefits in consequence of the working of the Insurance Act. Consequently the Distribution Committee had not spent so much money in this direction, and they had £1,000 which they would be able to refund, and thereby increase the grants to the participating institutions. The Chairman, having read the report of the Executive Committee *re* salaries of the official staff, it was resolved to defer its consideration to the next quarterly meeting. In the meantime a copy of the report, it was further agreed, should be forwarded to every member of the Board. It is interesting to note that the staff at present numbers fifteen, and includes a Secretary, £350 per annum; Organising Secretary, £200; Assistant Organising Secretary, £180; Cashier, £170; Assistant Cashier, £110; and a Confidential Clerk, etc. The approximate annual increase on the Fund, in the event of the Executive Committee's report being adopted, would be £100. Provided that their sum total bears a reasonably small proportion to the amount raised by the charities which they administer, to pay the officers well should be the basis of hospital administration.

### THE HOSPITAL COOK.

We have often been struck that one seldom hears complaints of the food (though sometimes of its service) supplied to patients in our hospitals. The average quality of the cooking must therefore, presumably, be high. Formerly there used to be many and just complaints by the nurses of the quality of the food and the character of the cooking supplied to them. Things have, however, improved greatly in this respect, and wherever nurses have cause to complain, there nowadays the matron and her housekeeper must be to blame. We came across a remarkable instance of long and efficient service at the Kent and Canterbury Hospital at Canterbury recently. On entering its kitchen we found there a cook who had occupied her position for thirty-three years, during the whole of which time the patients and everybody connected with the establishment had had good cause to be grateful to her for the excellent manner in which the food had been prepared and served. Fancy thirty-three continuous years of service in a hospital kitchen where the cooking had deservedly received the plaudits of patients and staff because the cook had a standard of personal conduct which she had diligently upheld under the encouraging influence of a great matron, Miss Messum, who has been matron at the Kent and Canterbury Hospital for twenty-seven years. We hope that the authorities at Canterbury will not fail to mark in a generous spirit the splendid services of their cook, and that her portrait may be permanently preserved somewhere within the hospital as an encouragement to those who come after. Will not some resident in each voluntary hospital throughout the country give the time to send us particulars of similar faithful service rendered by the humbler members of the permanent staff, seeing that long service for the sick in every department of a hospital is of supreme value, full of interest, and worthy of recognition?

### COTTAGE HOSPITALS AND MEDICAL STAFFS.

A strained situation which has arisen at Brentwood, in Essex, exemplifies a knotty point in institutional working which periodically causes heart-burnings amongst patients and doctors. The facts are that a Dr. Frazer, who has practised at Brentwood for twenty-five years, has certain patients for whom he considers treatment in hospital is desirable; and their cases are, in his opinion, suitable for the local Cottage Hospital.

The patients, however, will not entrust themselves to the Cottage Hospital unless they can remain under his care, and, as he is not one of the medical staff, this is impossible. He has applied for admission to the staff, but has been categorically refused. In a letter to the local press he states that ten years ago the Cottage Hospital was half its present size; but the staff then numbered five or six, whereas now it consists of three. He proceeds to caustic comments on this "nursing home for the clients of any particular firm, or firms, of surgeons," and on its status as an enterprise soliciting charitable subscriptions. The principle involved is, unfortunately, not easy to dogmatise upon; for the reasons that there is no hard-and-fast line of demarcation between a cottage hospital and a more ambitious institution, and that one not uncommonly expands into the other. But certainly it is desirable that in small country towns or villages cottage hospitals supported partly by public subscriptions should be open, as a rule, to all the qualified medical practitioners of the immediate vicinity. But the right of charities to select officers cannot be abrogated altogether, even in small cottage hospitals; and in larger institutions it is indispensable. It cannot, therefore, be said that all practitioners have an indefeasible right to cottage hospital appointments. But it is certainly arguable that grave reasons alone should justify exclusion.

### X-RAY CANCERS AND RADIATION THERAPY.

In a lecture before the Röntgen Society at the Middlesex Hospital on Monday, Dr. Jean Clunet, of Paris, described the upward growth of skin cells from within till cast off as dead scales. In cancers, he pointed out, the young cells did not age normally, but became active on their own account and multiplied. X-rays hastened the normal process, but a violent application produced a dead scale so quickly as to form an ulcer. Milder doses caused thinning and atrophy of the skin. He then explained that under strong rays the cells aged so quickly as to hinder their reproduction; very small doses stimulated their reproductivity. Thus the small doses to which the radiographer was subject caused inflammation on which cancer sometimes supervened. Moreover, *x*-ray cancers were peculiarly difficult to heal. In one case, doses large enough to be destructive were given, but as the nerves became affected the treatment had to be given up. The Director of the Cancer Research to the Middlesex Hospital declared that the paper marked a point in radiation therapy. It has certainly made clear the dangers and difficulties involved in this method of arresting cancer.

### THE WORKPEOPLE'S FUND AT LEICESTER.

The Leicester Royal Infirmary, well known for the generous support which the workpeople give to its funds, has received £500 from the workpeople of Messrs. N. Corah and Sons, of St. Margaret's Works. This is an increase of £100 on the contribution of the previous year. Messrs. Corah were pioneers in the introduction into Leicester of systematic contributions from the workpeople, and it is noteworthy that a sum of upwards of £8,700 has been contributed to the institution from the Fund, which has also lent generous support to other philanthropic local efforts. It is not surprising in the circumstances that the firm's donations from time to time have been as generous as their workpeople's.

### A CURIOUS SOURCE OF INCOME.

It is the essence of the voluntary system to make use of any and every source of income, and for this purpose to indoctrinate the public with the habit of giving to hospitals the first benefit of the doubt as to the disposal of any sums which they wish to employ for public purposes. An instance of this is seen at York, where the Recorder of Pontefract, Alderman Vernon Wragge, has given to the hospital £13, the natural history of which is somewhat curious. A year ago he promised twenty persons in York addicted to alcohol a sovereign each if for twelve months they were not once convicted of drunkenness. Of the twenty, seven succeeded and, with families or friends, were entertained to tea by him. Mr. Wragge further promised that he would add 25 per cent. to whatever sum remained "unearned" at the end of the year, together with any legitimate savings. Thus the, shall we say, unsuccessful abstainers' £13 has been handed to the hospital. At the tea Mr. Wragge offered to give these unlucky persons another chance on the same conditions, and added that whatever sum was not received by them should be divided equally between those of the seven successful abstainers who remained unconvicted. We trust that none of them will thereby be tempted to lure his fellows to excess. Convictions for the twenty fell from an average of about 70 to 27 last year.

### THIS WEEK'S DRUG MARKET.

As was expected, an advance in the price of santonin was definitely announced at the beginning of the year; a fair business was done prior to the advance. Apart from this one instance there had been hardly any noteworthy changes in a very quiet week. The price of iron and quinine citrate is slightly higher. Sugar of milk seems likely to advance in value. Citric acid is inclined towards somewhat lower prices, but no substantial decline is looked for at present. It is expected that oil of sandalwood will be dearer before long. The value of opium is well maintained and morphine is rather dearer. Cod-liver oil still tends lower in price in the absence of business. Natural benzoic acid (manufactured from gum benzoin) is rather dearer. There has been little change in the position of menthol, the price of which is less than one-third the figure quoted about a year ago; it is difficult to say what the tendency of prices will be in the near future, but at present there seems to be no sign of an impending advance. Cocaine is very quiet, but in view of the low prices now quoted it might not be altogether inadvisable to buy; a further decline in value is, of course, not impossible, but any material reduction is hardly to be expected.

